# The Perception and Estimation of Others' Pain according to Children

**DOI:** 10.1155/2016/9097542

**Published:** 2016-07-17

**Authors:** Mathieu Grégoire, Rosée Bruneau-Bhérer, Karine Morasse, Fanny Eugène, Philip L. Jackson

**Affiliations:** ^1^École de Psychologie, Université Laval, Québec City, QC, Canada G1V 0A6; ^2^Centre Interdisciplinaire de Recherche en Réadaptation et Intégration Sociale, Québec City, QC, Canada G1M 2S8; ^3^Centre de Recherche de l'Institut Universitaire en Santé Mentale de Québec, Québec City, QC, Canada G1J 2G3; ^4^Centre Intégré Universitaire de Santé et de Services Sociaux de la Capitale-Nationale-Centre Jeunesse de Québec (CIUSSSCN-CJQ), Québec City, QC, Canada G1C 3S2; ^5^CSSS Alphonse-Desjardins, Hôtel-Dieu de Lévis, Lévis, QC, Canada G6V 3Z1

## Abstract

Accurate interpretation of pain expressed by others is important for socialization; however, the development of this skill in children is still poorly understood. Empathy for pain models propose two main components (affective and cognitive), which develop at different stages of life. The study's objective was to investigate the children's ability between 3 and 12 years of age to detect and assess the pain intensity in others using visual stimuli depicting either facial expressions of pain or hands in painful contexts. 40 preschool children and 62 school-aged children were recruited. Children observed series of stimuli and evaluated the pain intensity depicted. Results demonstrated that children as young as three years old were able to detect and assess pain in both types of stimuli and this ability continued to improve until the age of 12. Participants demonstrated better detection performance with hands than with faces. Results were coherent with the idea that the two types of stimuli presented recruit different processes. Pain detection in hands appears to rely mostly on affective sharing processes that are effective early in life, while older children's higher ability to perceive pain in facial expressions suggests that this ability is associated with the gradual development of cognitive processes.

## 1. Introduction

Accurate detection and estimation of pain in others may be crucial to reacting appropriately, such as escaping to avoid a threatening situation or helping a person in pain. Although pain is often verbally expressed, many pain signals are transmitted through nonverbal cues, such as facial expressions, which are known to convey the majority of information needed to detect pain in others [1].

The development of pain perception in others starts early in human life. However, how the different processes underlying this ability interact throughout development is still unknown. Children as young as 14 to 18 months of age appear to be able to understand how others feel when they get hurt, and they react to others' distress with prosocial behaviours such as trying to help or comfort the person in pain [2]. A study by Deyo et al. [3] suggests that children are able to infer pain from adults' facial expressions by the age of five or six years, and this ability has been found to improve at least until the age of 12 years. Other cues, such as bodily damage, can also inform the observer about the intensity and potential unpleasantness of someone else's pain [4–6]. While children can decode facial expressions of pain at as early as five years of age [3], it is not yet clear at what age they start being able to infer pain in others from cues such as potentially painful situations or verbal descriptions. A recent study demonstrated that children between three and five years of age could recognize most common painful events shown in drawings [7]. Another report showed that children (four to seven years of age) were able to imagine verbally presented painful situations and evaluate their intensity [8]. These two types of stimuli require a certain knowledge and level of abstraction to be understood. In these studies, children as young as three years of age were found to have the necessary knowledge or experience to imagine the amount of pain related to some situations. However, there is a need for a better understanding of the kind of cues children use to understand and react to others' pain. For example, their ability to infer pain from pictures of limbs in painful situations is still unknown, despite the fact that this type of stimulus is frequently used, along with facial expressions stimuli, in sociocognitive studies examining pain perception in adults and adolescents.

The ability to recognize and understand others' physical and mental states, such as pain, is fundamental for socialization [9]. This ability relies on processes involved in empathy, which is defined as a response that stems from the understanding of another's state through the interaction of cognitive and affective processes and which predisposes to prosocial behaviours [10]. To explain this complex phenomenon, several functional models of empathy have been proposed [11–13], which are typically composed of two main components: affective sharing and mentalizing processes. Mentalizing has been defined as the ability to make inferences about the mental state of others [14]. It has been suggested that while automatic affective sharing is stable throughout childhood, the refinement of cognitive functions enables older children to use mentalizing more than younger children to understand others' mental states [15].

In adults, different studies have shown strong activation of cerebral regions associated with mentalizing processes during the observation of facial expressions of pain [16]. Conversely, the observation of a limb (hand or foot) in a painful situation has been associated, in children and adults, with brain activity related to affective sharing such as in sensorimotor areas [7, 16–18]. This suggests that perceiving pain in painful situations relies on affective sharing, while perceiving pain in facial expressions relies on cognitive abilities. Neural activity during the observation of both facial expressions of pain and limbs in painful contexts has also been associated with self-reported dispositional empathy in adults [19], which also supports the formulation that the development of pain detection and estimation abilities could be associated with the development of empathic traits. However, the link between the development of children's empathic traits and their ability to detect and estimate pain in others is still not well described.

## 2. Objectives and Hypotheses

The main objective of the present study was to investigate the ability of children between three and 12 years of age to detect pain in others using visual stimuli depicting facial expressions of pain or hands in painful contexts. Hypotheses were based on differences in the development of the affective and cognitive processes believed to underlie pain detection, pain estimation, and empathy. Because detecting pain in facial expressions is believed to rely mostly on cognitive processes such as mentalizing, pain detection in these stimuli was expected to improve with children's age. Conversely, because the detection of pain in painful situations is believed to rely more on affective sharing, which is believed to develop early and remain stable throughout childhood, pain detection performances involving the stimuli depicting limbs in painful contexts were not expected to vary with participants' age. A secondary objective of the present study was to investigate whether children could distinguish between different intensities of pain in others (pain estimation) when age-appropriate methods were used and whether this ability improves with age. It was hypothesized that older children would be better than younger children at fine-grained estimation of different levels of pain intensity in facial expressions. Lastly, the third objective was to determine whether perception of pain in the two types of stimuli would be differentially associated with the affective and cognitive components of dispositional empathy. It was hypothesized that pain intensity perceived in stimuli depicting hands in painful contexts would be positively correlated with the affective component of dispositional empathy and that pain intensity perceived in facial expressions would be positively correlated with its cognitive component.

## 3. Methods

### 3.1. Participants

Forty typically developing preschool children, three to five years of age, and 62 school-aged children, six to 12 years of age, were recruited from three public daycare facilities and three primary schools in Quebec City (Quebec) area. Inclusion criteria were as follows: to be between 36 and 71 months of age or to be in the first, third, or sixth grade at the time of the experiment to ensure a good distribution of grade school and to be proficient enough in French to understand the task. Based on parents' reports, none of the children presented an intellectual disability, previous neurological damage, noncorrected visual or hearing disabilities, chronic pain, or a diagnosed or suspected developmental disorder. Preschool children were pooled in one group and school-aged participants were separated into three groups based on their grade (first, third, or sixth). See Table 1 for demographic data. Parents' written consent and children's assent were obtained before the study. Children were given a small gift (preschool) or gift certificate (value of $10) for their participation. The present study was approved by the local ethics committees.

### 3.2. Material

#### 3.2.1. The Interpersonal Reactivity Index [20]

This 28-item questionnaire assesses four different aspects of empathy: perspective taking, fantasy, empathic concern, and personal distress. The first two aspects have been associated with the cognitive dimension of empathy, while empathic concern and personal distress have been associated with the questionnaires emotional dimension [21]. The original version of this scale was self-reported and asked participants to rate different items by providing a number between 0 (if an item does not describe “them” well) and 5 (if the item describes “them” very well). A French translation, previously used with an adult population, was adapted for parental report of children's abilities [22]. To ensure that parents were able to reliably estimate their children's empathic abilities, a pilot study was first performed in which adolescents' and young adults' (69 participants between 13 and 24 years of age) scores on the self-reported Interpersonal Reactivity Index (IRI) were compared with those provided by one of their parents on this newly developed parent-reported version. Results showed that this version of the IRI was sensitive enough to discriminate between more and less empathic children and that parents' scores closely matched those of their children [22].

#### 3.2.2. Pain Estimation Task

The task consisted of pseudodynamic visual stimuli depicting hands in painful or nonpainful situations (hands) and faces showing expressions of pain or neutral expressions (faces). E-Prime 2.0 (Psychology Software Tools, USA) was used to control stimulus presentation and to record answers. Each stimulus was composed of three ensuing digital pictures presented in sequence for durations of 1000 ms, 200 ms, and 1000 ms, respectively, to give the impression of movement and, therefore, produce a pseudodynamic clip [18]. The order of stimulus presentation was pseudorandomized following a rule to avoid >3 consecutive stimuli of the same category. Each stimulus was presented only once during the task. Because of their different cognitive abilities, different rating procedures were used for preschoolers and school-aged children. An adapted version of the Pieces of Hurt Tool [23] was used for preschoolers, and an 11-point numerical rating scale (NRS-11) was used with children between six and 12 years of age.

#### 3.2.3. Hands

Eighty stimuli depicting hands in different situations were selected from those used in a previous empathy study involving children [18]. One-half of the stimuli showed right hands in different painful situations (e.g., crushed by a car door), while the other one-half showed right hands in neutral situations (e.g., hand holding a doorknob). In all pictures, hands were partially masked using a Gaussian filter to reduce identification of sex and age of the model.

#### 3.2.4. Faces

Thirty-two stimuli showed adult faces with neutral or painful expressions. These pseudodynamic clips were extracted from 1 s long films involving facial expressions of pain produced by actors (four women and four men) asked to present neutral expressions (no pain condition) and four increasing levels of pain from low to extreme pain. These stimuli were developed and validated by Simon et al. [24].

### 3.3. Procedure

Children were met individually in a quiet room at their daycare or grade school for approximately 25 min. They sat in front of a portable computer and received instructions before the task. They were told that they were going to see individuals on the screen that would sometimes be hurt, sometimes not. They were also told that one hand of the person would be visible sometimes, but not always, and that they would sometimes have to guess whether the person was in pain based on their facial expression. To make sure they understood the concept of different intensities of pain, participants were given instructions on how to rate the intensity of the pain.

Preschool children were shown how to use the Pieces of Hurt Tool; three plastic chips were presented and children were told that these represented bits of pain. They were shown one chip and told it was a little pain, and then they were asked to recall a moment when they had experienced a little pain. Next, preschoolers were shown two chips and told that this represented more pain. Finally, they were shown three chips and told this was a lot of pain, and they were asked to recall a moment when they had felt a lot of pain. Then, children were told that for the test the chips would be on the computer screen. For the school-aged children, instructions for the NRS-11 were given before the task. The extreme labels (i.e., no pain and worst imaginable pain) were identified, and children were asked to recall a situation when they experienced a little pain and another one with a lot of pain and to show how they would evaluate those situations on the scale with a number between 0 and 10.

After the instructions and for all groups including preschoolers, four practice trials were conducted and repeated if necessary until all four trials were performed successfully to confirm that they understood the task. Each trial began with a prompt screen with the instructions, which were also orally presented by the experimenter. Each trial consisted of a 1500 ms fixation cross followed by the stimulus (hands or faces; 2200 ms total for the three pictures) and was manually started when the participant was looking at the screen. For preschoolers, a blank screen appeared after the stimulus and the children had to say whether the person was hurt or not. If the child said “no,” the next prompt screen appeared. If the child said “yes,” another screen appeared with a picture showing a single chip on the left, two chips in the middle, and three chips on the right of the screen. Children had to show (tell out loud or point on the screen) the level of pain observed in the stimulus before the next trial began. For school-aged children, the NRS-11 appeared on the screen immediately after the stimulus and the child answered orally with a number between 0 and 10. The preschoolers' and the school-aged children's responses were recorded on a keypad by the experimenter. For all children, short breaks were taken when needed to maximize their attention, and each child had at least one 5 min break between the two task blocks. Stimuli were pseudorandomized in each of the two blocks; however, the type of stimulus (hands and faces) and the sex of the actor (faces) were counterbalanced across blocks.

### 3.4. Data Analysis

The first objective of the present study was to document children's ability to perceive pain in two different types of stimuli (hands and faces). For each participant, hit rates and mean intensity ratings were calculated for each stimulus category. Shapiro-Wilk's normality tests showed that the normality assumption was violated; therefore, nonparametric tests were used to compare groups and types of stimuli.

Hit rates expressed as a percentage of correct answers (in school-aged children: ratings of 0 for neutral and >0 for painful stimuli) were used to compare detection performances for faces and hands stimuli within each group using Wilcoxon tests for repeated measures. To assess the impact of developmental level, mean hit rates for all observations together, as well as mean hit rates for hands and faces separately, were compared across groups using Kruskal-Wallis tests for independent samples. Mann-Whitney tests for independent samples, with the Bonferroni correction for multiple comparisons, were used to identify which groups significantly differed from each other.

The second objective of the present study was to assess the children's ability to distinguish between different pain intensities (pain estimation). In the present study, an NRS-11 was used with children as young as six years of age; however, the minimum age at which most children can accurately report pain on this type of scale is still unknown. Children ≤5 years of age have a limited grasp of series and numeracy; they tend to use only the extreme points on the scale [25, 26]. However, this tendency has never been tested in six- and seven-year-old children. In order to control for the high likelihood of biased responses anticipated in young children [26], bias analyses were realized. Bias analyses were performed using a Java program adapted from the version used by von Baeyer et al. [25], and each participant's response set was divided into 12 sequences of nine responses and tested separately for adherence to specific patterns. The mean percentage of biased sequences for each group was calculated.

Due to the use of different rating methods for preschoolers (Pieces of Hurt Tool) and school-aged children (NRS-11), comparisons among the mean pain ratings for each category (faces or hands), condition (pain or no pain), and pain level for faces (no pain, low, medium, high, or extreme pain) were conducted separately for each group. Differences among categories and conditions were tested using Wilcoxon tests for each group and the Friedman test for matched samples was used to compare ratings across pain levels. The Bonferroni correction was applied when multiple comparisons were made. Significant differences among the mean pain ratings for faces stimuli showing low, medium, high, and extreme pain would indicate that children can distinguish variations in pain intensities, which was our secondary objective. Finally, as the ability to perceive and to evaluate pain in both stimulus types was believed to relate to the maturation of empathic processes, Spearman correlations were used to test the potential link between pain intensity ratings and parent-reported dispositional empathy. Results from the IRI were first pooled into an affective component score (average of empathic concern and personal distress scores) and a cognitive component score (average of perspective taking and fantasy scales). These scores were each correlated with pain intensity ratings provided for the hands and faces stimuli separately.

## 4. Results

### 4.1. Rejection and Bias Rates

Because the main objective of the present study was to determine whether typically developing children can detect the presence or absence of pain in others, initial statistics evaluating pain detection were performed using the entire sample (*N* = 102). To ensure that analyses of intensity ratings included only children who were able to correctly detect the presence of pain, participants were excluded from subsequent analyses if their mean rating for pain stimuli was equal or inferior to their mean rating for the no pain stimuli in the hands and/or faces conditions or their hit rate was inferior to 50% for the hands or faces stimuli. It appears reasonable to assume that these children misunderstood the task, answered randomly, or could not detect pain in those stimuli (see Discussion). Based on these criteria, data from 12 participants were excluded from the remaining analyses (*N* = 90). Exclusion rates were low: preschoolers (15%), first graders (10%), third graders (10%), and sixth graders (9%). These low exclusion rates indicate that most children tested were able to correctly detect the presence of pain. Demographic data before and after exclusions are presented for each group in Table 1.

For school-aged children who used the NRS-11, bias analysis was performed with regard to anchors, middle, and sequence biases. Results demonstrated a higher percentage of biased performance patterns (all types combined) in the first-grade group (28%) than in the third-grade (16%) or sixth-grade groups (21%), which did not differ from each other. However, none of the groups' bias rates were significantly different from the adults' mean bias rate (21%) using a Bonferroni corrected alpha of 0.008 (0.05/6). The children's bias rates, therefore, appeared to be representative of normality and were not expected to influence critical variables in the present study.

### 4.2. Pain Detection: Differences between Stimulus Categories (All Children, *N* = 102)

**Figure 1:**
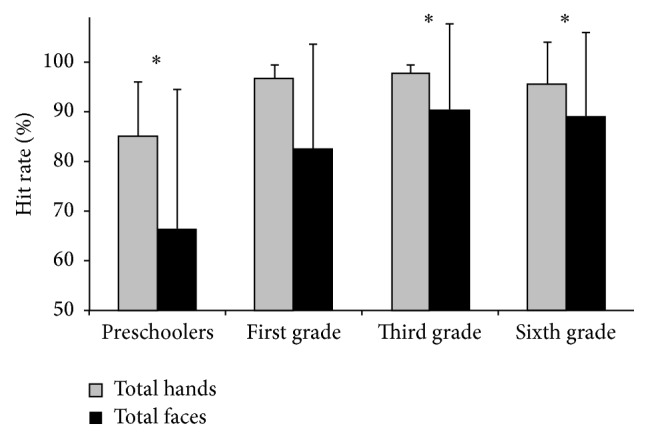
Mean hit rates for visual stimuli depicting faces showing expressions of pain or neutral expressions (faces) and hands in painful or nonpainful situations (hands) for each group. Error bars represent 1 SD. Significant differences (alpha level of 0.05) are indicated by an asterisk.

Mean hit rates were compared between stimulus categories (hands versus faces) within each group. Results indicated significantly higher hit rates for hands than for faces in preschoolers (*Z* = −3.448) and first and sixth graders (*Z* = −3.491 and *Z* = −2.091, resp.) at a 0.05 alpha level of confidence. The third-grade group showed only a marginally significant difference among hit rates, which were also higher for hands than for faces (*Z* = −1.751; *P* = 0.08). Cohen's *d* was calculated to characterize the magnitude of these differences across groups; preschool and first-grade groups presented large effect sizes (0.89 and 0.93, resp.) while medium effects were found for third- and sixth-grade groups (0.66 and 0.52, resp.).  Figure 1 illustrates the mean hit rates according to stimulus category for each group.

### 4.3. Pain Detection: Differences among Groups (*N* = 102)

Mean hit rates for all stimuli (faces and hands) were compared across groups, which yielded a significant difference among groups (*χ* ^2^  [3, *N* = 102] = 34.070; *P* < 0.05). Using a Bonferroni corrected alpha of 0.008, hit rates were found to be significantly lower in the preschool group than in the first-, third-, and sixth-grade groups (*P* < 0.008). No significant difference was found among the three school-aged groups' hit rates (*P* > 0.008 for all).

Group differences were also examined for faces and hands stimuli separately, which yielded significant group differences for hands (*χ* ^2^  [3, *N* = 102] = 44.911; *P* < 0.05) and for faces (*χ* ^2^  [3, *N* = 102] = 20.210; *P* < 0.05). For hands, the Mann-Whitney test for independent samples showed that preschoolers' hit rates were significantly lower than the three other groups' hit rates (*P* < 0.008). For faces, the Mann-Whitney test for independent samples revealed that preschoolers obtained significantly lower hit rates than the third- and sixth-grade groups (*P* < 0.008), and a similar, but insignificant, trend was found for the difference between the preschool and first-grade groups (*P* = 0.06). No significant differences were found among the first-, third-, and sixth-grade groups' hit rates for either hands or faces (*P* > 0.008 for all).

Note that the exclusion criterion based on detection performances and described previously was applied from this point on and, for the results described in the following sections, the total sample size was *N* = 90 (preschool [*N* = 34], first grade [*N* = 18], third grade [*N* = 18], and sixth grade [*N* = 20]). Because the groups used different rating scales, subsequent results are presented separately for preschoolers and school-aged children.

### 4.4. Preschoolers: Mean Pain Ratings (*N* = 34)

In preschoolers, mean (SD) pain ratings (range 0 to 3) attributed to hands stimuli were significantly higher in the pain (1.93 ± 0.47) than in the no pain (0.23 ± 0.30) condition (*Z* = −5.087; *P* < 0.05), confirming that they were able to discriminate between painful and neutral situations.

**Figure 2:**
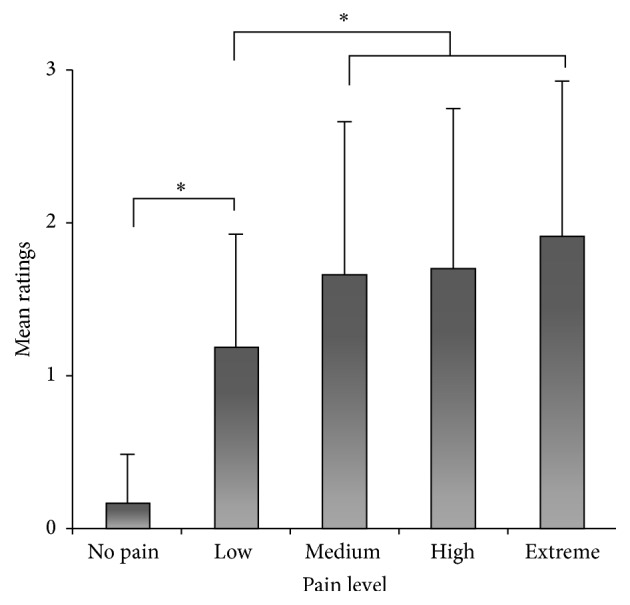
Preschoolers' mean pain ratings attributed to the different pain intensity levels for visual stimuli depicting faces showing expressions of pain or neutral expressions. Error bars represent 1 SD. Significant differences (*P* < 0.005) are indicated by an asterisk.

A Friedman test for paired samples showed a significant difference among the mean pain ratings attributed to faces stimuli for different pain intensity levels (no pain, low, medium, high, and extreme pain) (*χ* ^2^  [4, *N* = 34] = 73.63; *P* < 0.05). Using a Bonferroni corrected alpha of 0.005 (0.05/10), ratings for no pain stimuli were found to be significantly lower than ratings for every other intensity level (*P* < 0.005 for all), confirming that preschoolers can discriminate between the absence and presence of pain in facial expressions. Additionally, ratings for low pain stimuli were significantly lower than ratings for medium, high, and extreme pain (*P* < 0.005 for all), but ratings for medium, high, and extreme pain did not differ significantly from each other (*P* > 0.005 for all).  Figure 2 shows the mean pain ratings attributed to the different pain intensity levels for the faces stimuli.

### 4.5. School-Aged Children: Mean Pain Ratings (*N* = 56)

The mean (SD) pain ratings for hands stimuli in the first-, third-, and sixth-grade groups were 6.44 ± 2.20, 7.21 ± 1.21, and 6.06 ± 1.74, respectively, for the pain condition and 0.13 ± 0.17, 0.13 ± 0.16, and 0.09 ± 0.18, respectively, for the no pain condition. Mean ratings were significantly higher for the pain than for the no pain condition based on the Wilcoxon test for related samples (*Z* = −3.724, *Z* = −3.724, and *Z* = −3.92, resp., for the first-, third-, and sixth-grade groups; *P* < 0.05 for all), confirming that they were able to discriminate between painful and neutral situations.

In each group, a Friedman test for paired samples revealed significant differences in mean pain ratings attributed to the faces stimuli among pain levels (no pain, low, medium, high, and extreme pain): *χ* ^2^  (4, *N* = 18) = 59.643, *P* < 0.05 for the first-grade; *χ* ^2^  (4, *N* = 18) = 60.827, *P* < 0.05 for the third-grade; and *χ* ^2^  (4, *N* = 20) = 70.312, *P* < 0.05 for the sixth-grade group. In all groups, multiple comparisons corrected with a Bonferroni alpha of 0.005 (0.05/10) showed that ratings for no pain were significantly lower than those for every other level, and ratings for low pain were significantly lower than those for medium, high, and extreme pain (*P* < 0.005 for all). In the first-grade group, when applying a corrected alpha level (0.05/4; *P* = 0.025), there were no significant differences between ratings for medium and high pain or between ratings for high and extreme pain (*P* > 0.025 for all). All other comparisons were significant (*P* < 0.005 for all). A slightly different pattern was found in the third- and sixth-grade groups: ratings for high pain and extreme pain stimuli were not significantly different from each other (*P* = 0.047), but all other comparisons were significant (*P* < 0.005 for all).

**Figure 3:**
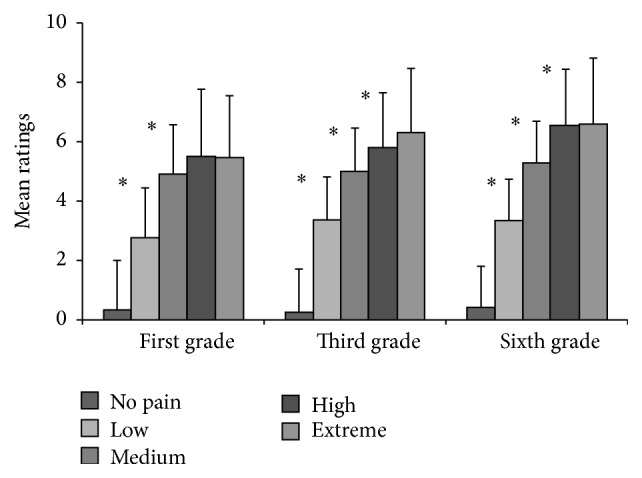
School-aged participants' mean pain ratings attributed to the different pain intensity levels for visual stimuli depicting faces showing expressions of pain or neutral expressions according to age group. Error bars represent 1 SD. Significant differences (*P* < 0.005) are indicated by an asterisk.

This indicates that children between six and 12 years of age were able to discriminate between low, medium, and high pain facial expressions and were able to report different pain intensity levels using an NRS-11.  Figure 3 illustrates the mean pain ratings attributed to the different pain levels for the faces stimuli for each group.

### 4.6. School-Aged Children: Empathy and Pain Estimation

Results on the IRI questionnaire were obtained for 38 school-aged children because a large number of parents did not send back the questionnaire or sent it with missing data. None of the correlations tested between pain intensity ratings (hands or faces) and scores on the affective and cognitive dimensions of empathy measured by the IRI were significant (*P* > 0.05 for all).

## 5. Discussion

The present study was designed to examine and compare the ability of children three to 12 years of age to detect and evaluate the intensity of pain in facial expressions and in stimuli depicting hands in painful contexts. The results revealed a high ability to detect pain in preschool years, which increases in school-aged children for both types of stimuli. They also showed that children were better at detecting pain in stimuli showing hands in painful contexts than in stimuli showing facial expressions of pain. Finally, all children were able to discriminate and report distinct pain intensity levels; however, the fact that we used different scales for preschool and school-aged children prevented us from comparing them directly. Overall, the findings suggest that children are able to detect and evaluate pain observed in others at a very young age and that this ability improves with the development of cognitive abilities.

### 5.1. Pain Detection and the Development of Empathy Components

Results from the present study suggest that the development of pain detection abilities in children follows a predictable trajectory consistent with the maturation of specific cognitive and affective processes that are also involved in empathy such as mentalizing and affective sharing. Most of the children involved in the present study had the ability to detect pain, although older children were better than preschoolers regardless of the type of stimulus used. This is consistent with results from Deyo et al. [3], who reported an increase in pain detection performance for facial expressions in children between five and 12 years of age. Results of the present study add to these findings by suggesting that this developmental trajectory begins earlier in life and by showing how it may vary according to the type of stimulus presented.

The developmental model of empathy proposed by Decety and Meyer [12] in 2008 was based on empirical evidence regarding the processes underlying the perception of pain and empathy for pain. The model is comprised of different neurocognitive components, which develop at different stages of life and are believed to influence children's ability to detect and evaluate pain in others. The first component of empathy, called affective sharing, is a key mechanism underlying the development of empathy in healthy children [12], and some authors have suggested that it may even be innate [17, 27]. This is coherent with the high detection rates found in every group for the hands stimuli, which indicate that children between three and 12 years of age can efficiently detect pain in these stimuli. Significant differences were found between preschoolers and the three other groups, which could be related to better attentional abilities in school-aged children, leading to lower error rates in this group.

Results also indicate that all groups were better at detecting the presence of pain in the hands stimuli than in the faces stimuli, as indicated by higher hit rates. Those two stimulus categories have different visual cues and appear to recruit different processes. A previous study suggested that when observing a limb in a painful situation, affective sharing is the main mechanism underlying the instant and automatic perception of pain in others [16]. This process is believed to develop early in life. Conversely, the perception of pain in facial expressions has been associated with cognitive processes such as mentalizing [16], a cognitive ability that relies on executive functions such as cognitive control and attention, which develop throughout childhood and adolescence [28]. Mentalizing is, therefore, believed to develop later than affective sharing, which is consistent with the present study's findings that children detected pain more easily in hands than in faces stimuli and that this difference was larger for preschoolers and first graders than for older children.

Results from the present study also showed that although all groups demonstrated an ability to distinguish between absence and presence of pain in the hands stimuli, school-aged children were significantly better at this task than preschoolers. This could be explained by the increased availability older children have to cognitive strategies, which can be used to extrapolate pain from the contextual information present in the hands stimuli. In fact, mentalizing processes include the ability to use contextual information to infer others' mental states in a given situation [26]. Although affective sharing is the main process believed to underlie pain perception in the hands stimuli, the development of mentalizing skills may, therefore, also improve the detection of pain from stimuli showing limbs in painful situations [29]. This is consistent with Decety and Meyer's formulation [12] that optimal pain estimation in others combines mentalizing and affective sharing processes.

Another factor that could have affected the detection rates for the faces stimuli in the present study is that younger children may have confused pain expressions with other negative emotions such as anger or fear (although the study was explicitly presented as being related only to pain). Adults have been demonstrated to clearly discriminate among the pain expressions used in the present study from other emotional expressions and from neutral expressions [25]; however, because it has been suggested that children acquire emotion categories gradually between the ages of two and five years [30], it is possible that some of the younger children were not yet proficient at this. It is also possible that the results of the present study may have been influenced by the use of adults' rather than children's faces, although studies suggest that children and adults show homologous muscular changes when expressing pain [25, 31]. However, as young children may be more often exposed to their peer's pain expressions, it is possible that different results could be obtained with stimuli in which facial expressions were concordant with the age of the participants.

### 5.2. Estimation of Pain Intensity

Because intellectual abilities vary greatly throughout childhood, the use of adapted measurement tools was necessary in the present study. The secondary objective was to investigate whether children can make accurate judgments of pain intensity based on facial expressions when age-appropriate methods are used and whether this sensitivity is related to developmental level. Results for preschoolers revealed a significant difference between two intensity levels (low and medium to extreme pain). For the third and sixth graders, the significant differences among the three different levels of pain indicate their increased ability to discriminate among pain intensities when compared with preschoolers. The first-grade group performed in a similar way as older children, although weaker differences were found. This intermediate performance pattern could represent a step in the natural development of the ability to perceive different pain intensities or in the ability to report such intensities on pain estimation scales.

Recent reviews involving children's self-report of their own pain intensity suggest that different measurement methods can be useful depending on the child's developmental stage [27, 32–35]. NRSs with a range between 0 and 10 (NRS-11), such as the one used in the present study, have been used successfully with children >7 years of age [8, 36]. For three- to four-year-olds, there is no standard tool to evaluate self-pain intensity; however, von Baeyer [34] suggests that the Pieces of Hurt Tool [23] is the best one available. In the present study, the use of the Pieces of Hurt scale with preschoolers with only three possible answers could have explained why they were only able to discriminate between two levels of pain compared with three in older children. However, this result is not related to scale saturation because the mean intensity rating, as presented in Figure 2, was <2 on a three-point scale. It shows, however, that preschoolers can discriminate between low pain intensity and medium to extreme pain intensities in facial expressions. Once there is substantial pain in the facial expression, preschoolers do not discriminate between higher pain levels even if the scale enables them to. This suggests that their estimation was not associated with a ceiling effect on the rating scale but with an absence of differentiation between higher pain levels, which could be interpreted as a poorer ability in pain level discrimination. Despite efforts made to adapt methodologies to children's intellectual abilities, the stage of development at which children can efficiently use scales for pain report is still unknown [34, 35].

The response bias or anchor bias reflects dichotomous thinking and the tendency for children to simplify complex tasks. The sequence bias or the propensity to select the first item on the scale to answer the first question and the subsequent item for the second one, in ascending or descending order, is also common. These response patterns in preschool children have been experimentally tested with regard to pain estimation with a Faces Pain Scale [25]. In the same study, it was found that age was negatively correlated with the presence of bias in responses in a group of three- to five-year-olds [25]. In the present study, bias analyses showed that 28% of the first-grade, 16% of the third-grade, and 21% of the sixth-grade groups presented anchor or sequence biases when reporting pain on the NRS-11, rates that were not significantly different from those of adults from a different experiment (Bruneau-Bhérer and Jackson, unpublished data; 25 adults aged 19 to 31 years). The lower percentage of participants showing biased response patterns in older groups (third and sixth grade) compared with the first-grade group is likely to be related to intellectual maturation.

The absence of correlation between scores on the empathy questionnaire and pain intensity ratings should be interpreted carefully because only a small portion of parents returned the questionnaire and, therefore, the negative results could be, in major part, attributed to the resulting small sample size. This result should, therefore, be replicated with a larger sample before drawing any definitive conclusion regarding the link between dispositional empathy and pain perception in children. Additionally, while it is possible to divide the IRI subscales into affective and cognitive components, the abilities measured by dispositional empathy questionnaires do not neatly map onto the cognitive and affective empathic processes believed to underlie pain detection and estimation, such as mentalizing and affective sharing [37]. Finally, some of the children in the current study were much younger than those with whom the parent-reported version of the IRI was initially validated (≥13 years of age), and it is possible that younger children's empathic abilities are more difficult for parents to assess accurately. Further validation of the parent-reported version of the IRI with different age groups is, therefore, warranted.

## 6. Conclusion

Overall, the results of the present study suggest that children three to five years of age can detect pain in others, are able to discriminate between two different levels of pain intensities in facial expressions, and can report their perceived pain intensities on a Pieces of Hurt instrument. As for children between six and 12 years of age, they can discriminate up to three different pain intensities using an NRS-11. Therefore, children between three and 12 years of age can perceive and evaluate pain experienced by others. This ability is proposed to rely mostly on affective sharing in preschool years, which is gradually combined with mentalizing abilities, consistent with cognitive development. Also, the use of different methodologies for different ages enables children to report the differences they perceive in pain intensities. In addition to improving our understanding of sociocognitive development, the present study may provide cues to improve the use of pain assessment tools in pediatric clinical settings. In fact, assessing the accuracy of children's estimation of their own pain has proven difficult [34]. The use of indirect measures of pain intensity through observation of other people's pain could provide a means of training children to use assessment tools, which could improve communication of their own pain, a crucial step in optimal pain care.

## Figures and Tables

**Table 1a:** (a) Sociodemographic information for each group, before exclusion (*N* = 102).

	Groups			
	Preschool	First grade	Third grade	Sixth grade
Sample size (*N*)	40	20	20	22
Number of females (%)	17 (43)	14 (70)	11 (55)	11 (50)
Mean age in months (SD)	51 (9.31)	86 (4.66)	111 (4.91)	147 (4.69)
Age range in months	36–68	72–92	104–124	142–156

**Table 1b:** (b) Sociodemographic information for each group, after exclusion (*N* = 90).

	Groups			
	Preschool	First grade	Third grade	Sixth grade
Sample size (*N*)	37	18	18	20
Number of females (%)	16 (43)	12 (67)	9 (50)	10 (50)
Mean age in months (SD)	52 (9.50)	86 (4.83)	111 (4.82)	147 (4.75)
Age range in months	36–68	72–92	104–124	142–156
